# The Signature Emotions of a Happy Life, a Meaningful Life, and a Psychologically Rich Life

**DOI:** 10.1007/s42761-026-00393-6

**Published:** 2026-07-31

**Authors:** Shigehiro Oishi, Youngjae Cha

**Affiliations:** Department of Psychology, University of Chicago, 5848 S. University Ave, Chicago, IL 60637 USA

**Keywords:** Well-being, Emotion, Happiness, Meaning, Psychological richness

## Abstract

In two studies, we examined the signature emotions of three aspects of a good life, using multiple data analytic techniques ranging from Lasso and Elastic net regressions to bootstrapping-based stability analyses of predictor selections to Bayesian Adaptive Sampling (BAS)-based posterior inclusion probability. In Study 1 (*N* = 158), a happy life (measured by life satisfaction) was best captured by happiness, satisfaction, and the relative lack of regret and suffering. A meaningful life was characterized by hope and passion. A psychologically rich life was captured by passion, delight, grief, and lack of dejection. In Study 2 (*N* = 435), a happy life was again best characterized by happiness, satisfaction, and lack of regret. A meaningful life was best captured by hope, passion, enjoyment, and the relative lack of unhappiness and hate. A psychologically rich life was signified by thrill, compassion, grief, and the relative lack of hate, unhappiness, and embarrassment. Across two studies, happiness, satisfaction, and the relative lack of regret were signature emotions of a happy life, whereas hope and passion were signature emotions of a meaningful life. Finally, delight/thrill and grief were signature emotions of a psychologically rich life. These distinct sets of signature emotions help advance our understanding of emotional experiences specific to happiness, meaning, and psychological richness.

Decades of subjective well-being (SWB) research have focused almost entirely on broad dimensions of positive versus negative emotions (Diener et al., 2018), leaving the emotional texture of a good life largely unmapped at the discrete level. The main goals of this research are (1) to identify signature emotions for three aspects of a good life: a happy life, a meaningful life, and a psychologically rich life (Oishi & Westgate, 2022, 2025), using 100 discrete emotions, and (2) to provide more nuanced understanding of the emotional tone of a good life, beyond broad dimensions of positive affect (PA) versus negative affect (NA).

## Emotions in Subjective Well-Being

Subjective well-being researchers have considered emotional experiences as a central indicator of self-reported well-being (Diener, 1984) to such an extent that some researchers include the presence of positive emotions and the relative absence of negative emotions as part of the definition of SWB (Diener et al., 2018). The frequency of PA is associated with higher levels of SWB, whereas the frequency of NA is associated with lower levels of SWB (Busseri, 2018). Similarly, PA is associated with higher levels of Ryff’s (1989) six dimensions of psychological well-being (PWB), whereas NA is associated with lower levels of PWB (Anglim et al., 2020).

## Discrete Emotions and Well-Being

Emotion researchers have long debated the relative advantages of the dimensional approach, which focuses on the higher-order dimensions such as valence and arousal (Barrett, 1998; Larsen & Diener, 1992; Russell, 1980) versus the discrete emotion approach, which focuses on discrete emotions such as happiness, pride, and awe (Cowen & Keltner, 2017; Ekman, 1994; Fredrickson, 2004; Shiota et al., 2017). Many emotion researchers used both approaches to investigate antecedents and consequences of emotional experiences (e.g., Harmon-Jones et al., 2017). In contrast, well-being researchers took the dimensional approach as the default and rarely used the discrete emotion approach. Because of this historical fact, we will first contrast the dimensional approach versus the discrete approach in the related area of personality psychology.

### Why Discrete Emotions?

A large meta-analysis (Anglim et al., 2020) found that extraversion and conscientiousness were fairly similarly correlated with positive affect (PA: extraversion *r* = .44 vs. conscientiousness *r* = .35) and negative affect (NA: extraversion *r* = − .21 vs. conscientiousness *r* = − .25). Openness to experience and agreeableness were also similarly associated with PA (openness *r* = .24 vs. agreeableness *r* = .19). However, these correlations might have obscured more nuanced emotion-personality associations. Indeed, Shiota et al. (2006) found that extraversion was most strongly correlated with joy, love and pride (see also Izard et al., 1993), whereas conscientiousness was most strongly associated with pride and contentment. Because extraversion is a more interpersonal trait (Eid et al., 2003) than conscientiousness (which is associated with self-regulation, Eisenberg et al., 2014), it makes sense that love captures extraversion more than conscientiousness. Similarly, Shiota et al. found that agreeableness was most strongly associated with compassion and love, whereas openness to experience was most strongly associated with awe and compassion. Again, it makes sense that compassion and love are the signature emotions of agreeableness, given that it is associated with kindness and tenderness (Graziano & Eisenberg, 1997), and that awe, an aesthetic emotion (Keltner & Haidt, 2003), is the signature emotion of openness to experience, which is by definition a behavioral tendency toward aesthetic experience.

In short, although three aspects of a good life are all highly associated with PA (Oishi & Westgate, 2022), different discrete emotions are likely to signify these three aspects of a good life. One study examined 14 discrete emotions experienced among older adults, and found that happiness, contentment, and gratitude were positively associated with self-reported life satisfaction (hope, pride, and relief were not), while boredom, sadness, frustration, fear, and regret were associated with lower levels of life satisfaction (guilt and shame were unrelated, Chipperfield et al., 2003). Another study found that those high in life satisfaction spontaneously described their situation/feelings using the word categories such as happy, glad, and respect more frequently, while using the word categories such as boredom, fear, anger, and sadness less frequently (Song & Zhao, 2023). Thus, life satisfaction should be characterized by happiness, gladness, satisfaction, and contentment, and the absence of unhappiness, sadness, boredom, fear, anger, and suffering.

Considering that meaning in life is gained by having a strong sense of purpose, future orientations (Baumeister et al., 2013), and commitment to family and community (Steger et al., 2006), discrete emotions such as hope, passion, and compassion should be its signature emotions. Indeed, Edwards et al. (2025) found that hope was a unique and robust predictor of meaning in life above and beyond PA. Finally, given that a psychologically rich life is defined as a life filled with diverse, unusual experiences that entail perspective change (Oishi & Westgate, 2022, 2025), discrete emotions that indicate (a) adventure, risk, and novelty such as excitement and thrill and (b) intense engagement such as passion and enthusiasm might be its signature emotions.

In sum, we conducted two studies to explore signature emotions of a happy life, a meaningful life, and a psychologically rich life out of 100 most prototypical emotions from Shaver et al.’s (1987) 213 emotion words. To identify a small number of signature emotions, we utilized Lasso (Least Absolute Shrinkage and Selection Operator) regression, which is particularly suitable for selecting robust predictors out of many predictors (McNeish, 2015).

## Study 1

### Method

#### Participants

Participants were 158 students (47 men, 105 women, 6 others) at the University of Virginia who participated in our study in exchange for partial course credit. Of these, 99 self-identified as White, 15 as Black, 12 as Latinx, 45 as Asian, 1 as Pacific Islander, and 2 as others. The mean age of the participants was 19.23 (*SD* = 1.49). This study was approved by UVa’s IRB-SBS.

### Materials and Procedures

After signing a consent form, they completed an online survey, which included the Satisfaction with Life Scale (SWLS; Diener et al., 1985, omega = 0.92), a measure of a happy life, the presence subscale of the Meaning in Life Questionnaire (MLQ-P; Steger et al., 2006, omega = 0.90), a measure of a meaningful life, and the 17-item version of the Psychologically Rich Life Questionnaire (PRLQ; Oishi et al., 2019, omega = 0.95), a measure of a psychologically rich life.

Then, they completed the 100-item emotion scale that we created based on Shaver et al.’s (1987) prototypicality ratings (100 emotion terms that were rated highest on prototypicality of emotion, 2.99 to 3.94 on the 1–4 point prototypicality rating where 1 was “I would definitely not call this an emotion” and 4 was “I would definitely call this an emotion”). The 100 emotions included in the survey were love, anger, hate, depression, fear, jealousy, happiness, passion, affection, sadness, grief, rage, aggravation, ecstasy, sorrow, compassion, joy, envy, fright, terror, elation, guilt, excitement, anguish, embarrassment, worry, panic, unhappiness, anxiety, desire, horror, sympathy, shame, lust, disgust, hostility, jubilation, loneliness, delight, pleasure, tenderness, pity, bitterness, disappointment, humiliation, dejection, despair, frustration, hurt, adoration, agony, thrill, fury, remorse, agitation, outrage, resentment, dislike, glee, alienation, distress, enjoyment, relief, gloom, misery, euphoria, bliss, gladness, regret, rejection, pride, gaiety, homesickness, jolliness, nervousness, woe, longing, loathing, satisfaction, hope, abhorrence, insecurity, defeat, dread, fondness, enthusiasm, sentimentality, hopelessness, annoyance, cheerfulness, displeasure, melancholy, glumness, shock, spite, suffering, dismay, exasperation, infatuation, and apprehension. Participants were asked to indicate how much they felt each emotion during the past month (1 = never or very rarely, 2 = rarely, 3 = sometimes, 4 = often, 5 = very often or always).

Both Studies 1 and 2’s data, R codes, and outputs are available at https://osf.io/9dj6b/overview?view_only=d38a0e6ce5cd45c2be72b19074da562e.

## Results and Discussion

In order to identify signature emotions for each form of a good life, we implemented a multi-stage analytic pipeline designed to assess the consistency of results across complementary frameworks, starting with Lasso and Elastic net regressions to test relative predictive contribution under different penalizations (L1 penalty in Lasso, L1 + L2 penalty in Elastic net), followed by resampling-based stability assessment, and Bayesian model-based uncertainty estimation. Specifically, we first conducted penalized regression analyses using Lasso and elastic net models implemented in the *glmnet* package in R. These approaches were used to obtain sparse predictive representations of the 100 emotions. Lasso regression reduces overfitting relative to unregularized OLS by imposing an L1 penalty that shrinks coefficients toward zero (Tibshirani, 1996). We report Lasso models using the 1SE rule (Lasso 1se), which yields more parsimonious solutions than the minimum cross-validation error criterion (Bainter et al., 2023).

Because Lasso solutions can be sensitive to multicollinearity among predictors, we additionally ran elastic net models, which combine L1 and L2 penalties and are better suited for handling correlated predictors by encouraging grouped selection. Third, to evaluate the stability of predictor selection, we conducted bootstrap stability selection analyses (100 resamples), estimating the frequency with which each predictor was selected across resampled datasets. This allowed us to assess the robustness of Lasso-and elastic net-based selections under sampling variability.

Finally, we calculated a posterior inclusion probability (PIP) for each emotion, using Bayesian Adaptive Sampling (BAS), with the package *bas* in R (Clyde et al., 2011). PIP indicates how likely each emotion belongs in the true model across all plausible models with all different combinations of predictors (i.e., under model uncertainty). Thus, this final analysis gives us information regarding how necessary each emotion is across different models. Thus, BAS-based PIPs provide very different information than the first 4 analyses, which are frequentist and more focused on stability across subsamples rather than inclusion probability under diverse prediction models (i.e., different combinations of 1 predictor models, 2 predictors models, 3 predictors models etc.). Overall, this analytic strategy aimed to identify emotions that are consistently associated with each aspect of a good life across multiple penalized regression models, resampling-based stability assessments, and Bayesian posterior inclusion probabilities.

### A Happy Life

We first tested which of the 100 emotions would be associated with life satisfaction. Lasso 1se analysis retained 9 emotions, while elastic net analysis retained 12 emotions. Table 1 shows 9 emotions that had the largest coefficients in both analyses. As seen in Table 2, Lasso 1se analysis with 9 emotions accounted for 48.8% of variance, whereas the elastic net analysis with 12 emotions accounted for 49.1% of variance in SWLS.

When predictors are correlated (case in point here), predictor selections could be unstable (Bainter et al., 2023). In order to formally test the stability (replicability) of the Lasso and Elastic net results, we then ran the stability of predictor selection analysis, using a bootstrapping method (100 times), which provided information regarding percentage of times each emotion was selected in re-runs of Lasso and elastic net analyses with 100 subsamples. This told us how stable each predictor is across 100 different subsamples. Table 1 includes 9 emotions that were retained by Lasso 1se and the additional emotions retained by Elastic net if they had stability coefficients above 0.70. Highlighted in bold in Table 1 are emotions with non-zero Lasso and Elastic net coefficients with the stability coefficients above 0.70 and BAS PIP above 0.50.

In terms of stability (how often each emotion was selected in analyses with subsamples), regret was most frequently selected (88%), followed by suffering (86%). The stability analysis with 100 resamples on Elastic net analyses showed that regret was again most frequently chosen (91%), followed by suffering (85%).

Finally, we calculated a posterior inclusion probability (PIP) for each emotion, using Bayesian Adaptive Sampling (BAS). Higher PIPs indicate that these emotions are plausible members of the predictive models (Gelman et al., 2013). As seen in Table 1, regret had the highest PIP (0.944), followed by happiness, suffering, satisfaction, and infatuation. In sum, Lasso 1se, elastic net, two stability analyses and BAS analysis consistently showed that happiness, satisfaction, and the lack of regret and suffering are the signature emotions for a happy life.

### A Meaningful Life

We repeated the same five analyses for meaning in life. Both Lasso 1se and elastic net analyses resulted in only two emotions selected: hope and passion (Table 1). As seen in Table 2, the Lasso 1se model with the two emotions accounted for 29.0% of variance, while the elastic net model with the same two emotions accounted for 28.9% of variance in meaning in life. The stability analysis showed that hope and passion were both included in 96–99% of subsampling analyses, suggesting a remarkably high degree of robustness. In addition, cheerfulness and the lack of hate and hopelessness appeared fairly consistently, though Lasso 1se and elastic net analyses deemed their coefficients virtually zero, indicating very small associations with meaning in life. Finally, BAS analysis showed that hope and passion are almost definitively part of the most predictive models. Across all five analytic approaches, results consistently indicate that hope and passion are the signature emotions of a meaningful life.

### A Psychologically Rich Life

Finally, we repeated the same 5 analyses on a psychologically rich life. Lasso 1se analysis resulted in 10 emotions, whereas elastic net analysis resulted in 8 emotions (Table 1). As seen in Table 2, the Lasso 1se model with 10 emotions accounted for 35.4% of variance, whereas the elastic net model with 8 emotions accounted for 32.4% of variance in psychological richness. Both Lasso 1se and elastic net analyses found passion, delight, and enthusiasm to be the top 3 predictors of a psychologically rich life, followed by tenderness, the lack of dejection, hope, the lack of regret, and the presence of grief. Interestingly, grief was a positive predictor of a psychologically rich life in 88–93% of the bootstrapping samples (the most consistent predictor) and had the second highest posterior inclusion probability (75.6%) in BAS. Collectively, passion, delight, grief, and the lack of dejection are the signature emotions of a psychologically rich life.

In sum, Lasso 1se, elastic net, two stability selection analyses, and BAS revealed different signature emotions for the three types of a good life. A happy life was mainly captured by happiness, satisfaction, and lack of regret and suffering. A meaningful life was characterized by hope and passion, the two emotions that capture future orientations and dedication. A psychologically rich life was captured by passion, delight, grief, and the lack of dejection, a diverse collection of intense interpersonal emotions.

These findings need to be interpreted with some caution, as there are two major limitations: (a) the study’s modest sample size, which makes these estimations less reliable, and (b) college student participants, who limit generalizability. For instance, some of these emotions such as hope and passion might be particularly salient among young achievement-oriented participants but might not be among older adults. These limitations are addressed in Study 2.

**Table 1:** Lasso regression and elastic net (enet) coefficients with bootstrapping stability indices and Bayesian Adaptive Sampling (BAS) Posterior Inclusion Probability (PIP) for each type of a good life in Study 1

Emotion	Lasso_coef	Enet_coef	Lasso	Enet	BAS PIP
A Happy Life					
1 **Happiness**	**0.241**	** 0.174**	0.61	**0.76**	** 0.563**
2 **Regret**	** -0.136**	** -0.102**	** 0.88**	** 0.91**	** 0.944**
3 Delight	**0.130**	** 0.106**	** 0.73**	** 0.84**	0.129
4 **Satisfaction**	** 0.090**	** 0.075**	0.62	**0.70**	** 0.518**
5 **Suffering**	** -0.085**	** -0.062**	** 0.86**	** 0.85**	** 0.665**
6 Depression	-0.056	-0.056	0.46	**0.72**	0.083
7 Enjoyment	0.023	0.064	0.34	0.52	0.020
8 Unhappiness	-0.016	-0.029	0.51	0.52	0.016
9 Love	0.016	0.017	0.69	**0.73**	0.019
10. Infatuation	0.000	0.000	**0.72**	** 0.78**	** 0.549**
A Meaningful Life					
1 **Hope**	**0.234**	**0.184**	**0.96**	** 1.00**	** 0.994**
2 **Passion**	**0.153**	**0.112**	**0.96**	**0.97 **	**0.967**
3 Hate	0.000	0.000	**0.73**	0.65	0.115
4. Hopeles	0.000	0.000	0.68	0.69	0.251
5. Cheerfulness	0.000	0.000	**0.75**	0.69	**0.524**
A Psychological Rich Life					
1 **Passion**	**0.148**	**0.124**	**0.73**	**0.81**	**0.864**
2 **Delight**	**0.080**	**0.073**	**0.71**	**0.85**	0.384
3 Enthusiasm	0.073	0.064	0.61	0.65	0.180
4 Tenderness	0.040	0.025	**0.79**	**0.84**	0.062
5 **Dejection**	**-0.035**	**-0.017**	**0.75**	**0.81**	**0.514**
6 Hope	0.032	0.031	0.37	0.48	0.056
7 Regret	-0.026	-0.010	0.55	0.69	0.086
8 **Grief**	**0.018**	**0.000**	**0.88**	**0.93**	**0.756**
9 Pride	0.018	0.000	0.47	0.60	0.054
10 Shame	-0.002	0.000	0.53	0.71	0.215
11 Defeat	0.000	0.000	**0.71**	**0.75**	0.051

Note. In bold are the emotions that showed strong predictive values under two regularization models that also were deemed stable across subsamples (over 0.70) and highly probable to be included in the final predictive model (over 0.50)

### Study 2

We conducted Study 2 to address the two major limitations of Study 1, namely whether Study 1 findings could be replicated and generalizable to non-college student adults in the U.S.

## Method

### Participants

A total of 509 participants were recruited through Cloud Research Connect. To screen out AI-generated or inattentive responses, we administered an instructed-response item in which two short lines of text were displayed and participants were asked to type only the highlighted word (“fiSH”), exactly as shown and with matching capitalization; 69 participants who provided an incorrect or blank response were excluded. In addition, at the end of the survey, participants completed a self-report data-quality item (“Do you believe that your data should be included in analysis?“; 1 = Yes, 2 = No). No participant selected No, but 5 participants with missing responses on this item were excluded. The final analytic sample comprised 435 participants (210 women, 48.3%): 320 self-identified as White (73.6%); 39 (9%) as Black; 13 (3%) as Asian; 51 (11.7%) as Latinx; 10 (2.3%) as Multi-race; 2 (0.5%) as others. Their ages ranged from 18 to 81 (*M* = 45.86, *SD* = 16.16). This study was approved by the University of Chicago’s IRB.

### Materials and Procedures

Participants completed an online survey. A happy life was again assessed with SWLS (Diener et al., 1985, omega = 0.92). A meaningful life was again assessed with the presence subscale of the Meaning in Life Questionnaire (Steger et al., 2006, omega = 0.94). A psychologically rich life was again assessed with the Psychologically Rich Life Questionnaire (Oishi et al., 2019, omega = 0.95). Then, participants indicated how frequently they experienced each of the same 100 emotions during the past month on the same 5-point scale used in Study 1.

**Table 2:** Fit indices (Mean Squared Error: MSE, Root Mean Squared Error, RMSE, and R^2^) of Lasso 1se and elastic net models

Model	MSE	RMSE	*R* ^2^	*N_predictors*
**Study 1**				
A Happy Life				
1 Lasso (1se)	1.043	1.021	0.488	9
2 Elastic Net	1.014	1.007	0.491	12
A Meaningful Life				
1 Lasso (1se)	1.048	1.024	0.290	2
2 Elastic Net	1.147	1.071	0.289	2
A Psych Rich Life				
1 Lasso (1se)	0.575	0.758	0.354	10
2 Elastic Net	0.578	0.760	0.324	8
**Study 2**				
A Happy Life				
1 Lasso (1se)	1.076	1.038	0.617	15
2 Elastic Net	1.090	1.044	0.616	18
A Meaningful Life				
1 Lasso (1se)	1.258	1.121	0.562	12
2 Elastic Net	1.231	1.109	0.570	17
A Psychologically Rich Life				
1 Lasso (1se)	0.883	0.940	0.472	24
2 Elastic Net	0.815	0.903	0.472	25

## Results and Discussion

### A Happy Life

We repeated exactly the same analyses on life satisfaction as in Study 1. Lasso 1se selected 15 emotions (*r*^*2*^ = 0.617), while elastic net model selected 18 emotions (*r*^*2*^ = 0.616). Table 3 shows Lasso and Elastic net coefficients, stability coefficients for both analyses, and BAS PIP. Included in Table 3 are 15 emotions selected by Lasso 1se plus terror, which received a very high PIP (0.980). Highlighted in bold are emotions with non-zero Lasso and Elastic net coefficients with the stability coefficients above 0.70 and BAS PIP above 0.50. Replicating Study 1, a happy life was again best characterized by happiness, satisfaction, gladness, and the lack of unhappiness and regret. In Study 2, loneliness was an additional signature emotion of a happy life, whereas suffering was not replicated as a signature emotion.

### A Meaningful Life

We repeated the same analyses on meaning in life. Lasso 1se selected 12 emotions (*r*^*2*^ = 0.562), whereas elastic net model selected 17 emotions (*r*^*2*^ = 0.570). Emotions included in Table 3 were 12 emotions selected by Lasso 1se plus 2 emotions that had high levels of stability coefficients (over 0.70) or BAS PIP (over 0.50). Replicating Study 1, hope again was a very robust predictor of a meaningful life. Passion, the other signature emotion in Study 1, was also a very reliable predictor across various subsamples (82% in Lasso, 91% in Elastic net). However, BAS PIP indicated that passion was a relatively weak predictor (recall BAS PIP indicates the probability of being included as a critical predictor in all plausible prediction models in Bayesian analyses, whereas stability analyses are being selected as a predictor in different subsamples of Lasso and Elastic net). In Study 2, enjoyment and the lack of unhappiness were also robust predictors of a meaningful life. In sum, a meaningful life was best characterized by hope, enjoyment, and the relative lack of unhappiness, with passion also emerging as a predictor in Lasso and Elastic net analyses.

### A Psychologically Rich Life

Finally, we repeated the same analyses on psychological richness. Lasso 1se model selected 24 emotions (*r*^*2*^ = 0.472), whereas the elastic net model selected 25 emotions (*r*^*2*^ = 0.472). Emotions included in Table 3 were 24 emotions selected by Lasso 1se plus 1 emotion that had high levels of stability coefficients (over 0.70) and high BAS PIPs (over 0.50). Overall, a psychologically rich life was most consistently characterized by thrill, compassion, and the relative lack of hate, embarrassment, and unhappiness. Of the Study 1 signature emotions (passion, delight, grief, and the lack of dejection), only grief was robustly replicated, with having extremely high BAS PIP (0.998) and stability coefficients (0.79 for Lasso, 0.84 for Elastic net). Overall, then, thrill, compassion, grief, and the relative lack of hate, embarrassment, and unhappiness are robust signature emotions of a psychologically rich life in Study 2.

**Table 3:** Lasso regression and elastic net (enet) coefficients with bootstrapping stability indices and Bayesian Adaptive Sampling (BAS) Posterior Inclusion Probability (PIP) for each type of a good life in Study 2

Emotion	Lasso_coef	Enet_coef	Lasso	Enet	BAS PIP
A Happy Life					
1 **Happiness**	**0.244**	**0.208**	**1.00**	**0.99**	**0.998**
2 **Satisfaction**	**0.219 **	**0.178 **	**0.99 **	**1.00**	** 0.987**
3 **Unhappiness**	**−0.205 **	**−0.169 **	**0.93 **	**0.98**	** 0.984**
4 Joy	0.121	0.121	**0.89 **	**0.87**	0.028
5 **Regret**	**−0.081 **	**−0.066 **	**0.91 **	**0.93**	** 0.690**
6 **Gladness**	**0.081 **	**0.080 **	**0.87 **	**0.84**	** 0.584**
7 Loneliness	−0.070	−0.065	**0.77 **	**0.89**	0.361
8 Love	0.056	0.048	**0.86 **	**0.97**	0.323
9 Relief	0.047	0.023	**0.84 **	**0.91**	0.023
10 Insecurity	−0.040	−0.035	0.63	0.64	0.028
11 Depression	−0.037	–0.047	0.68	**0.71**	0.007
12 Bliss	0.037	0.028	0.50	**0.75**	0.007
13 Disappointment	−0.033	−0.030	0.61	**0.74**	0.017
14 Elation	0.012	0.005	0.54	0.60	0.012
15 Hopelessness	−0.007	–0.018	**0.70 **	**0.80**	0.061
16 Terror	0.000	0.000	0.56	0.57	**0.980**
A Meaningful Life					
1 **Unhappiness**	**−0.243**	**−0.192**	** 0.95**	**0.98**	** 1.000**
2 **Hope**	**0.198**	**0.168 **	**1.00**	**1.00**	** 1.000**
3 Happiness	0.185	0.158	**0.96**	**0.95**	0.130
4 **Enjoyment**	**0.147**	**0.133 **	**0.94**	**0.96**	** 0.987**
5 Hopelessness	−0.078	–0.092	**0.95**	**0.95**	0.126
6 Satisfaction	0.073	0.077	**0.70**	**0.74**	0.014
7 Hate	−0.042	–0.068	**0.75**	**0.86**	0.272
8 Loneliness	−0.040	–0.053	0.51	0.67	0.046
9 Passion	0.035	0.067	**0.82**	**0.91**	0.103
10 Dejection	−0.024	–0.048	0.57	0.67	0.057
11 Cheerfulness	0.007	0.035	0.38	0.47	0.020
12 Enthusiasm	0.006	0.033	0.55	0.69	0.020
13 Love	0.000	0.019	0.58	**0.73**	0.033
14. Envy	0.000	–0.001	0.59	0.67	**0.506**
A Psychologically Rich Life					
1 **Hate**	**−0.096**	**−0.090**	**0.94**	**0.89**	** 0.783**
2 **Thrill**	**0.095**	**0.085**	**0.93**	**0.96**	** 0.999**
3 **Embarrassment**	**−0.086**	**−0.078**	**0.97**	**0.98**	** 0.999**
4 Love	0.084	0.077	**0.80**	**0.87**	0.040
5 Pleasure	0.071	0.063	0.65	0.69	**0.513**
6 **Unhappiness**	**−0.069**	**−0.062**	**0.76**	**0.78**	** 0.956**
7 Hope	0.066	0.059	**0.77**	** 0.83**	0.088
8 Tenderness	0.059	0.059	**0.77**	** 0.89**	0.013
9 **Compassion**	**0.058**	**0.054**	**0.83 **	**0.88**	** 0.973**
10 Gladness	0.046	0.043	0.50	0.62	0.092
11 Enthusiasm	0.042	0.041	0.63	**0.77**	0.018
12 Fondness	0.041	0.039	0.65	**0.73**	** 0.877**
13 Nervousness	−0.040	–0.040	**0.78**	** 0.81**	0.113
14 Enjoyment	0.035	0.037	0.53	0.63	0.206
15 Cheerfulness	0.028	0.031	0.45	0.51	0.008
16 Hopelessness	−0.022	–0.024	**0.70**	0.63	0.032
17 Delight	0.021	0.025	0.50	0.61	0.011
18 Jubilation	0.020	0.021	0.34	0.34	0.005
19 Ecstasy	0.018	0.018	0.55	0.54	0.019
20 Jealousy	−0.017	–0.021	0.55	0.53	0.005
21 Happiness	0.013	0.021	0.43	0.45	0.111
22 Passion	0.012	0.018	0.53	0.62	0.044
23 Adoration	0.011	0.016	0.48	0.53	0.003
24 Satisfaction	0.005	0.014	0.32	0.37	0.017
25 Grief	0.000	0.0000	**0.79**	**0.84**	** 0.998**

Note. In bold are the emotions that showed strong predictive values under two regularization models that were also deemed stable across subsamples (over 0.70) and highly probable to be included in the final predictive model: BAS PIP (over 0.50)

## General Discussion

In two studies, we sought to identify signature emotions of a good life. A happy life (measured by life satisfaction) was mainly captured by happiness, satisfaction, and the lack of regret and suffering in Study 1. Replicating Study 1, a happy life was again best characterized by happiness, satisfaction, and the lack of regret in Study 2. These results also serve as “sanity checks” for our analyses in that, logically speaking, a happy life should be signified by happiness, satisfaction, and the lack of regret.

A meaningful life was characterized by hope and passion in Study 1. A meaningful life was again best characterized by hope, whereas passion was a robust predictor in Lasso and Elastic net models, but a weak predictor in BAS in Study 2. A meaningful life was also robustly associated with enjoyment and the relative lack of unhappiness and hate in Study 2. Overall, hope was the most robust signature emotion of a meaningful life, followed by passion. These findings make sense in that a meaningful life is a life with a clear sense of future directions (Baumeister et al., 2013), and hope is a future-oriented emotion (Snyder et al., 1991). It is also noteworthy that our bottom-up approach conceptually replicated Edwards et al.’s (2025) theoretically derived findings on the link between hope and meaning in life. In addition, we found that passion was another signature emotion of a meaningful life. Passion signals deep engagement with the world and perseverance (Jachimowicz et al., 2018), which is likely to result in a positive societal impact. Among adult participants in Study 2, those leading a meaningful life might be deriving enjoyment from their societal contributions. Passion was a weaker predictor of a meaningful life in Study 2. This could be due to the tendency for young people to report passion more frequently than older adults. It is important to explore further potential developmental shifts in signature emotions of a meaningful life.

Finally, a psychologically rich life was captured by passion, delight, grief, and the lack of dejection in Study 1. In contrast, it was best characterized by thrill, compassion, grief, and the relative lack of hate, unhappiness, and embarrassment in Study 2. Grief was the only signature emotion from Study 1 that was highly stable across different subsamples and BAS models in Study 2. Overall, then, the signature emotions of a psychologically rich life varied across studies except for grief. However, delight in Study 1 and thrill in Study 2 are similar in intensity. The lack of dejection in Study 1 and the lack of unhappiness in Study 2 are also functionally equivalent. Signature emotions of a psychologically rich life appear to center on intense interpersonal emotions both positive and negative.

Characteristic experiences associated with psychological richness are adventures such as study abroad (Oishi et al., 2021), which are in turn captured by emotions such as delight and thrill. Grief is typically experienced as a result of losing someone important, and often encapsulates a complex series of events and changes in relationships and identity (Bonanno & Kaltman, 2001). Such complexity and changes might be a reason why grief is *positively* associated with psychological richness. Notably, grief was positively associated with psychological richness, whereas dejection, unhappiness, and embarrassment were negatively associated with it. It is important to explore why grief, unlike other negative emotions, was positively associated with psychological richness in the future.

Before concluding, some limitations should be recognized. First, although we measured 100 prototypical emotions from Shaver et al. (1987), some well-known discrete emotions were not included. For instance, awe, surprise, and interest that are part of Cowen and Keltner’s (2017) 27 discrete emotions were not included. It is important to explore signature emotions, using a different set of emotions that ideally include all major discrete emotions. Second, our studies were conducted in the U.S. Thus, our findings might be limited to the U.S. For instance, given Tsai and colleagues’ extensive research on cultural variations in affect evaluation (Tsai et al., 2025), low activation positive emotions such as calm and peace are likely to be signature emotions of a happy life among East Asians. It is important to document signature emotions of three aspects of a good life across diverse cultures in the future. Third, our studies relied on self-reports. It is critical to test whether similar emotions are retained as signature emotions, using non-self-reports.

In conclusion, Lasso/Elastic net regression analyses, two stability selection analyses, and BAS-based revealed three sets of signature emotions for a good life. Signature emotions of a happy life were happiness, satisfaction, and the lack of regret. Signature emotions of a meaningful life were hope and passion. Signature emotions of a psychologically rich life were delight/thrill and grief. Given the limitations of our methods and samples, these should be considered preliminary candidates for signature emotions for each aspect of a good life. We look forward to seeing more research exploring discrete emotions that signify each aspect of a good life to enrich our understanding of the crucial role emotions play in subjective well-being.

## Additional Information

### Availability of data and material

Data Availability Data for both Studies 1 and 2 are available at https://osf.io/9dj6b/overview?viewonly=d38a0e6ce5cd45c2be72b19074da562e.
